# Impact of Clinoptilolite and Anionic Salts on Calcium Homeostasis, Parathyroid Hormone, and Related Metabolic Parameters in Periparturient Dairy Cows

**DOI:** 10.3390/vetsci13050408

**Published:** 2026-04-22

**Authors:** Pengyu Huang, Xiu Su, Yuanyin Guo, Chong Ma, Jie Cao

**Affiliations:** College of Veterinary Medicine, China Agricultural University, Beijing 100193, China; h1956663036@163.com (P.H.); sy20243051209@cau.edu.cn (X.S.); guoyuanyin979@163.com (Y.G.)

**Keywords:** periparturient dairy cows, clinoptilolite, anionic salts, calcium homeostasis, metabolic changes

## Abstract

This study compared the regulatory effects of dietary supplementation with natural clinoptilolite (CLN) versus a dietary cation-anion difference (DCAD) regimen on calcium homeostasis in dairy cows during the last 21 days prepartum. Results showed that cows in the DCAD group exhibited significantly higher blood ionized calcium (iCa) and parathyroid hormone (PTH) concentrations than those in the CLN group (*p* < 0.05). Serum PTH concentrations displayed a declining trend in both groups prepartum, which deviates from the classical theory of compensatory PTH secretion, suggesting that alternative compensatory pathways may be involved in the regulation of calcium homeostasis during the periparturient period in dairy cows. Monitoring of calcium homeostasis and related metabolic parameters following postpartum oral calcium bolus administration revealed that the incidence of subclinical hypocalcemia in the DCAD group was 26%, lower than the 62% observed in the CLN group. However, blood iCa concentrations returned to normal levels more rapidly in the CLN group. Additionally, CLN supplementation was associated with more stable blood pH and lower prepartum serum potassium concentrations (*p* < 0.05) that remained within the physiological range, which may contribute to improved tissue sensitivity to PTH.

## Simple Summary

Postpartum hypocalcemia is a major metabolic disorder in dairy cows. Common prevention strategies include prepartum anionic salt diets, calcium-modified diets, and postpartum calcium supplementation. Comparing calcium homeostasis and metabolic status under different dietary regimens can facilitate the development of precise postpartum calcium supplementation protocols to support a smooth periparturient transition. Cows fed an anionic salt diet maintained higher prepartum blood ionized calcium and parathyroid hormone concentrations, but showed greater individual variation in postpartum blood ionized calcium. In comparison, cows fed an ultra-low-calcium diet with clinoptilolite exhibited lower postpartum blood ionized calcium concentrations, yet smaller individual variation, faster recovery of blood ionized calcium, and more stable blood acid–base balance and potassium status. The ultra-low-calcium diet with clinoptilolite avoids reliance on low-potassium forages, improves palatability, and exerts milder calcium regulatory effects, which favors a stable periparturient period. The dynamic changes in ionized calcium and parathyroid hormone indicate that other factors may be involved in the regulation of blood calcium homeostasis in postpartum dairy cows.

## 1. Introduction

During the periparturient period, dairy cows experience a sharp increase in calcium demand, and postpartum hypocalcemia is one of the most representative metabolic disorders. The incidence of subclinical hypocalcemia in multiparous cows can reach 50%, and it significantly elevates the risk of other diseases including ketosis, abomasal displacement, metritis, and mastitis [1]. However, hypocalcemia is also closely associated with acid–base balance, mineral metabolism, energy regulation, and the endocrine system [2].

Calcium homeostasis is primarily regulated by the parathyroid hormone (PTH)–vitamin D axis, and this process is also modulated by other physiological systems. Acid–base balance not only alters the ionization state of blood calcium—with ionized calcium (iCa) increasing by approximately 5.3% for every 0.1-unit decrease in blood pH [3]—but also influences tissue sensitivity to PTH [4]. Furthermore, shifts in acid–base status affect rumen microbiota, thereby altering the efficiency of nutrient digestion and absorption [5,6]. As the major intracellular cation, potassium (K^+^) not only maintains neuromuscular function but also competes with Ca^2+^ for reabsorption in the renal distal tubules. Elevated blood K^+^ concentrations may exacerbate urinary calcium loss [7,8]. Excessively high potassium content in forages can induce metabolic alkalosis in dairy cows, which in turn alters the three-dimensional conformation of PTH receptors in target organs such as bone and kidney. This reduces target tissue sensitivity and responsiveness to PTH, thereby exacerbating hypocalcemia [9].

Energy metabolism is closely linked to hypocalcemia [10]. Hypocalcemia inhibits the peristalsis of gastrointestinal smooth muscle, leading to reduced feed intake and thereby exacerbating negative energy balance (NEB), and elevates blood non-esterified fatty acid (NEFA) concentrations [11]. High levels of NEFA not only induce fatty liver and ketosis but also cause immunosuppression and predispose cows to pathogenic microbial invasion [12,13]. Lipopolysaccharide released from the death and lysis of Gram-negative bacteria that invade dairy cows triggers acute, transient hypocalcemia [14].

To prevent postpartum hypocalcemia, dairy farms commonly supplement anionic salts or zeolite in the diets of prepartum transition cows [15,16]. Anionic salts work by lowering the DCAD, inducing mild metabolic acidosis to boost calcium metabolism [17]. Zeolite additives offer a “non-acidifying” alternative. Synthetic zeolite A works largely by binding phosphorus in the gut, which indirectly activates the calcium system [18]. Natural clinoptilolite has a different structure (a silicon-to-aluminum ratio of about 5:1), making it more stable in acidic conditions [19]. Beyond calcium and phosphorus, clinoptilolite may affect rumen fermentation, nitrogen metabolism, and even immune function [20,21,22]. Despite these possibilities, there is limited research systematically comparing how clinoptilolite affects metabolic adaptation—especially key biomarkers—in transition cows.

This study aimed to systematically compare the effects of clinoptilolite versus anionic salt diets on blood iCa, PTH, blood glucose, pH, and K^+^ in periparturient dairy cows. It analyzed the divergent roles of the two strategies in related metabolic pathways during calcium homeostasis regulation, as well as their impacts on metabolic recovery within 24 h postpartum. We hypothesized that clinoptilolite improves calcium metabolism with milder effects on acid–base balance and other electrolytes compared with anionic salts. Such differences would be evident not only prepartum but also persist postpartum, affecting the rate and stability of metabolic recovery.

## 2. Materials and Methods

### 2.1. Experimental Design and Animals

The study was approved by the IACUC of China Agricultural University (Protocol AW51705202-2-201) and conducted in July 2024 at two comparable commercial dairy farms (>10,000 cows) in Hebei, China. Both farms followed identical standard operating procedures (SOPs) for management and milking to minimize farm-level confounding. The Dingzhou farm used the CLN diet, while the Hengshui farm used the DCAD diet. Cows were housed in free stalls with TMR fed at 07:00 and 19:00. Dietary composition is detailed in Table 1.

Prepartum Phase: 50 Holstein cows were selected from each of the two farms according to the following criteria: parity 2–3, 21 ± 3 days before expected calving, body condition score 3.25–3.5, and good health status. (1) CLN-Pre: basal diet + 500 g/d natural clinoptilolite (80-mesh; Baokin Mineral Products, Chengde, China); (2) DCAD-Pre: anionic diet (DCAD −10.7 mEq/100 g DM).

Postpartum Phase: 10 newly fresh multiparous Holstein cows were selected from each of the two farms and enrolled in the trial: the clinoptilolite–postpartum group (CLN-Post, *n* = 10) and the anionic salt-postpartum group (DCAD-Post, *n* = 10). All experimental cows were orally administered one calcium bolus (Bovikalc^®^, Boehringer Ingelheim, Ingelheim am Rhein, Germany) at 0 h and 12 h postpartum, respectively. Colostrum was collected within 1 h after calving.

### 2.2. Blood Sample Collection and Laboratory Measurements

#### 2.2.1. Sampling Time Points

Prepartum Phase: blood samples were collected from the cows via coccygeal venipuncture before they were fed in the morning on days −21, −14, and −7 relative to calving and immediately after parturition (0 h). Postpartum Phase: blood samples were collected at 0, 1, 6, 12, 13, and 24 h after calving.

#### 2.2.2. Sampling Procedure

Blood was collected from the coccygeal vein using heparinized tubes (2 mL for immediate blood gas analysis) and clot activator tubes (5 mL). Serum was separated by centrifugation at 3000 r/min for 10 min at 4 °C, then stored at −20 °C for PTH determination.

#### 2.2.3. Blood Gas, Electrolyte Measurements, and PTH Assay

Within 5 min of collection, a portable analyzer (i-STAT 300i, Abbott, Chicago, IL, USA; CG8+ cartridges) was utilized to measure glucose, iCa, K^+^, and blood pH. Serum PTH was measured using a bovine-specific ELISA kit (Cat. No. JL16416; Jianglai Biological Technology Co., Shanghai, China). According to the manufacturer’s protocol, both intra-assay and inter-assay CVs of this kit were below 10%.

### 2.3. Statistical Analysis

All computational procedures and hypothesis testing were executed within the Python environment (version 3.10.18). Specifically, data wrangling relied on the Pandas library (v2.3.2), while the Pingouin package (v0.5.5) was employed for inferential statistics. Significance thresholds were strictly established at α = 0.05. Normality assessments combined Shapiro–Wilk metrics with visual inspection of Quantile–Quantile (Q-Q) plots. Both groups demonstrated homogeneity of variance (Levene’s test, *p* > 0.05) and normality of distribution (Shapiro–Wilk test, *p* > 0.05). Regarding the prepartum dataset (*n* = 50), the application of parametric independent sample *t*-tests was deemed appropriate owing to the Central Limit Theorem, despite minor distributional skewness. Conversely, analysis of the smaller postpartum subset (*n* = 10) necessitated conservative interpretation. Evaluation of the two experimental phases was conducted independently. Prepartum longitudinal variables (−14 d, −7 d, 0 h) were modeled using Analysis of Covariance (ANCOVA), utilizing the −21 d baseline metric as a covariate to mathematically adjust for initial biological variability.

## 3. Results

### 3.1. Blood Ionized Calcium

#### 3.1.1. Effects of the Two Periparturient Diets on Blood Ionized Calcium

**Figure 1:**
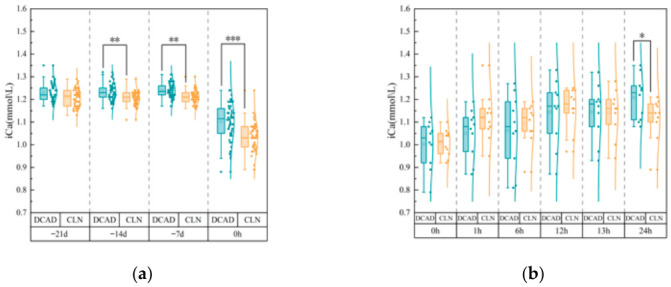
Changes in blood iCa concentrations in multiparous dairy cows administered different periparturient diets. (**a**) Prepartum iCa concentrations (−21 d to calving). (**b**) Postpartum iCa concentrations (0–24 h postpartum). All cows received oral calcium supplementation at 0 h and 12 h postpartum. Symbols: * *p* < 0.05, ** *p* < 0.01, and *** *p* < 0.001.

Prepartum Phase (Figure 1a): No significant difference in baseline iCa was found at the trial’s inception (Day −21). Blood iCa concentrations in the CLN group were significantly lower than those in the DCAD group at −14 d and −7 d prepartum (*p* < 0.01). Although a temporal reduction in systemic iCa occurred in both groups approaching parturition, a distinct inversion was recorded at the moment of calving (0 h); at this juncture, iCa retention in the DCAD cohort significantly exceeded that of the CLN group (*p* < 0.001).

Postpartum Phase (Figure 1b): Following parturition, an expected physiological nadir in blood iCa was universally observed at 0 h. Administration of oral calcium boluses triggered a restorative trend. Throughout the acute recovery window (1 h through 13 h postpartum), recovery trajectories remained statistically parallel between treatment arms (*p* > 0.05). Differentiation in calcemic status re-emerged at the 24 h postpartum mark, where the DCAD group maintained a statistically superior magnitude of recovery compared with the CLN cows (*p* < 0.05).

#### 3.1.2. Incidence of Hypocalcemia

Blood iCa concentrations ≤ 1.05 mmol/L were classified as subclinical hypocalcemia, and concentrations ≤ 0.85 mmol/L were classified as severe subclinical hypocalcemia, which is associated with a greater risk of periparturient paresis. The density distributions of iCa concentrations during the prepartum and postpartum periods are summarized in Table 2 and Table 3. Across the three prepartum monitoring points (−21 d, −14 d, and −7 d), no cows in either group showed signs of subclinical hypocalcemia. During calving (0 h), the incidence of subclinical hypocalcemia was 26% (13/50) in the DCAD group and 62% (31/50) in the CLN group. Severe subclinical hypocalcemia (iCa ≤ 0.85 mmol/L) was not observed in either group.

Among the 20 cows tracked after calving, the DCAD group had six cases of subclinical hypocalcemia at birth, with one cow showing severe hypocalcemia (iCa ≤ 0.85 mmol/L). In the CLN group, eight cows exhibited subclinical hypocalcemia, but none had iCa levels below the severe threshold of 0.85 mmol/L. We administered calcium orally at 0 h and 12 h postpartum. By 24 h, the blood calcium content of all cows in the DCAD group had recovered to normal levels, whereas two cows in the CLN group remained subclinically hypocalcemic.

**Figure 2:**
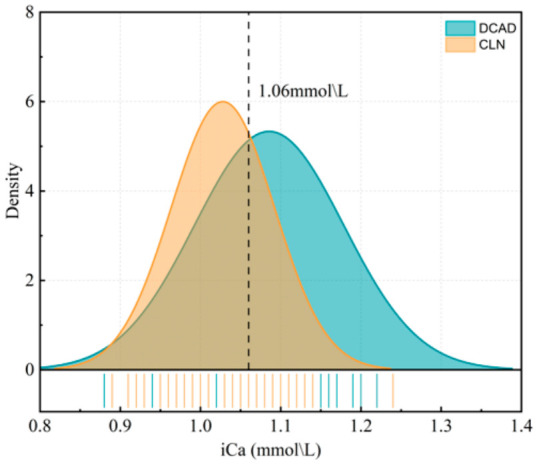
Density distribution of blood iCa concentrations at calving in cows administered the CLN diet (*n* = 60) or the DCAD diet (*n* = 60) during the prepartum period. The vertical dashed line at 1.06 mmol/L indicates the diagnostic threshold for subclinical hypocalcemia (<1.06 mmol/L).

Figure 2 combines the iCa data from all cows in both the prepartum and postpartum cohorts (*n* = 50 + 10) to visualize the difference at calving. The density plot shows a rightward shift for the DCAD group, indicating that the peak density occurred at a higher iCa concentration than that observed in the CLN group. This pattern indicates that a greater proportion of cows in the DCAD group maintained adequate blood calcium concentrations at calving, whereas more cows in the CLN group were near the lower limit of the normal range of blood calcium concentration (1.06 mmol/L).

### 3.2. PTH

**Figure 3:**
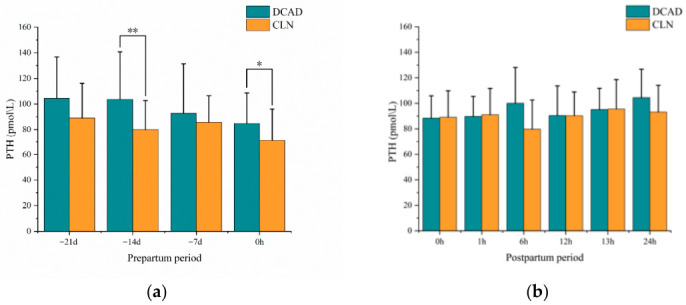
Serum PTH concentrations in cows administered the CLN or DCAD diet during the periparturient period. (**a**) Prepartum period. (**b**) Postpartum period. Symbols: * *p* < 0.05, ** *p* < 0.01.

During the prepartum period, the baseline serum PTH concentrations at −21 d did not differ significantly between the two groups (Figure 3a). At −14 d, cows in the DCAD group had significantly higher PTH concentrations than those in the CLN group (*p* < 0.01). No significant differences were observed at −7 d (*p* > 0.05). On the day of calving (0 h), PTH concentrations were again significantly greater in the DCAD group than in the CLN group (*p* < 0.05).

During the postpartum period, no significant differences in serum PTH concentrations were detected between the two groups throughout the monitoring period of 24 h (Figure 3b). The PTH concentrations were maintained within the normal physiological range in both groups, and the temporal fluctuations followed a similar pattern.

### 3.3. Blood pH, Potassium (K^+^), and Blood Glucose

**Figure 4:**
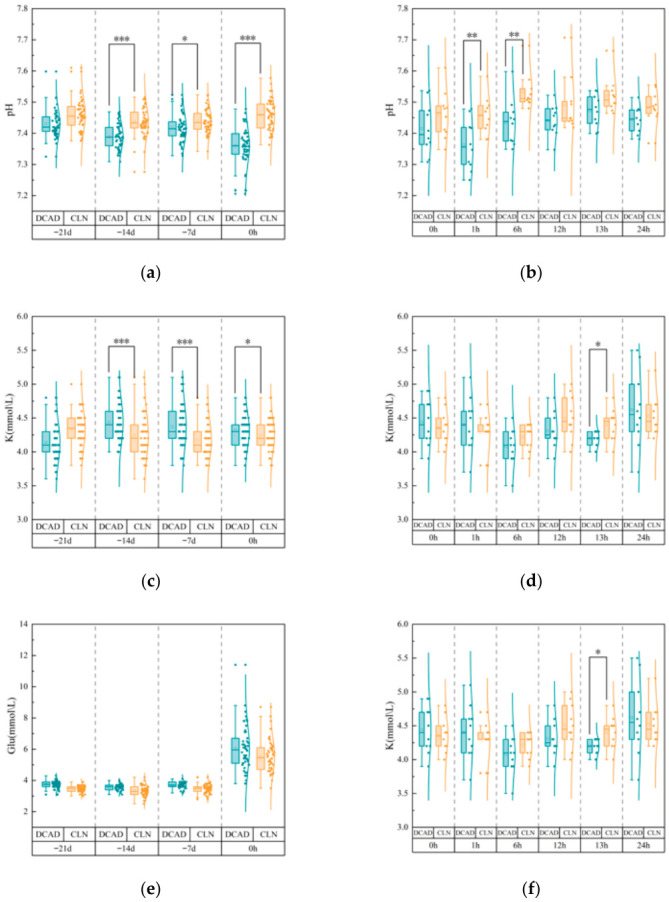
Blood pH, K^+^, and blood glucose in cows receiving the CLN or DCAD diet during the periparturient period. (**a**,**c**,**e**) Prepartum period. (**b**,**d**,**f**) Postpartum period. Symbols: * *p* < 0.05, ** *p* < 0.01, and *** *p* < 0.001.

**Figure 5:**
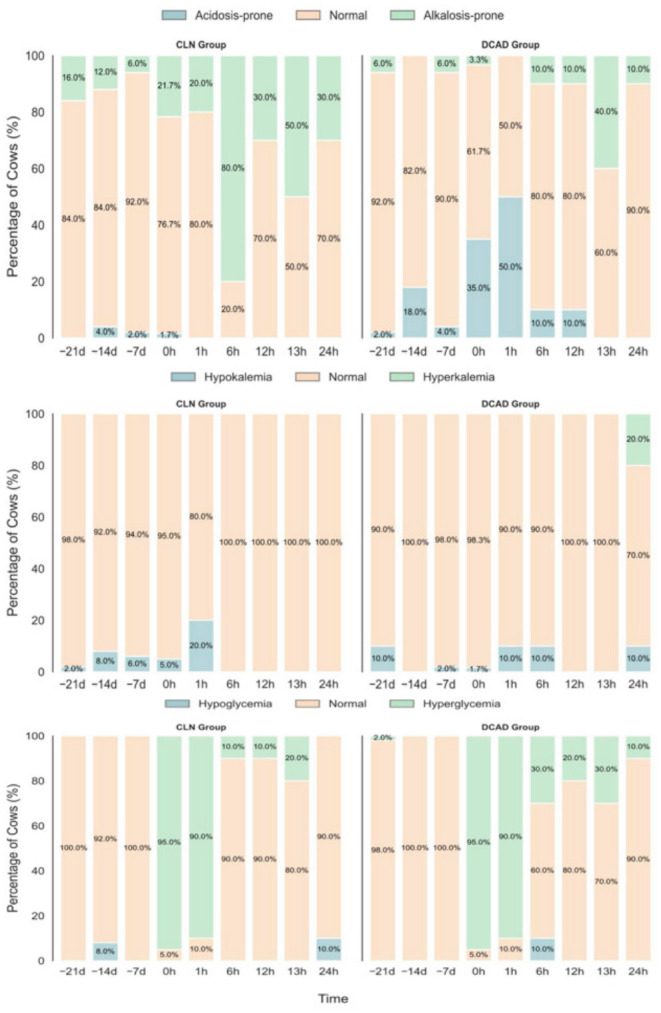
Distribution of metabolic status categories in cows receiving the CLN or DCAD diet during the periparturient period. From top to bottom, panels show the percentage of cows classified as: acidotic (pH < 7.35), normal (pH 7.35–7.50), or alkalotic (pH > 7.50) at each monitoring time point; hypokalemic (K^+^ < 3.9 mmol/L), normal (K^+^ 3.9–5.3 mmol/L), or hyperkalemic (K^+^ > 5.3 mmol/L); and hypoglycemic (Glu < 2.78 mmol/L), normal (Glu 2.78–4.28 mmol/L), or hyperglycemic (Glu > 4.28 mmol/L).

After baseline adjustment, the mean blood pH of cows in the DCAD group was significantly lower than that of those in the CLN group at −14 d (*p* < 0.001), −7 d (*p* < 0.05), and 0 h (*p* < 0.001) (Figure 4a). Consistent with this finding, the proportion of cows showing signs of acidosis (pH < 7.35) was markedly greater in the DCAD group (Figure 5), reaching 35.0% at 0 h postpartum compared to only 1.7% in the CLN group (*n* = 60). At 1 h and 6 h postpartum, the mean blood pH remained significantly lower in the DCAD group (*p* < 0.05), and at 1 h postpartum, half of the DCAD cows (50.0%) were in an acidotic state.

During the prepartum period, baseline-corrected blood K^+^ concentrations were significantly greater in the DCAD group than in the CLN group at −14 d, −7 d (both *p* < 0.001), and 0 h (*p* < 0.05). However, based on clinical categorization (Figure 5), >98% of the cows in both groups showed K^+^ values within the normal physiological range (3.9–5.3 mmol/L). During the postpartum period, blood K^+^ concentrations did not differ between groups at most time points, except at 13 h postpartum, when the CLN group presented lower K^+^ levels (*p* < 0.05). At 24 h postpartum, 20.0% of DCAD cows developed hyperkalemia (K^+^ > 5.3 mmol/L), whereas all cows in the CLN group had normal levels of K^+^.

Blood glucose concentrations did not differ between groups at any prepartum monitoring point (−14 d, −7 d, and 0 h). Similarly, during the postpartum phase, no significant group differences were detected during peak stress or during most of the recovery phase. Additionally, individual recovery patterns varied. From 6 h to 13 h postpartum, more than 80% of the cows in the CLN group maintained normal glucose values, whereas 20–30% of the DCAD cows exhibited hyperglycemia. At 24 h postpartum, 10.0% of DCAD cows remained hyperglycemic, whereas no cows in the CLN group were hyperglycemic; instead, 10.0% of CLN cows presented mild hypoglycemia.

## 4. Discussion

### 4.1. Regulation of Calcium Homeostasis

Previous studies demonstrated that when the dietary calcium content was maintained at about 0.78%, cows receiving zeolite supplementation presented higher serum calcium concentrations both prepartum and postpartum compared to cows in the control and anionic salt groups [18]. Frizzarini et al. proposed that zeolite A may enhance calcium homeostasis by lowering serum phosphorus, thereby relieving the inhibitory effect of hyperphosphatemia on the conversion of 25-hydroxyvitamin D_3_ to 1,25-dihydroxyvitamin D_3_, which subsequently improves the absorption of intestinal calcium. Additionally, zeolite A may promote bone calcium mobilization through the downregulation of fibroblast growth factor 23 (FGF23).

Since the calcium content of both diets had been long-term validated by the respective farms for effective prevention of postpartum hypocalcemia, dietary calcium levels were not standardized between the two groups in this study. We observed that blood iCa concentrations were significantly higher in the DCAD group than in the CLN group during the prepartum period. We speculate that this difference was mainly attributed to the higher dietary calcium level in the DCAD diet (0.70% vs. 0.49%). Furthermore, the anionic salt diet induced metabolic acidosis, which in turn increased blood iCa concentrations, whereas clinoptilolite bound part of the calcium in the intestinal tract, further reducing calcium absorption in the CLN group. These prepartum iCa results should be interpreted with caution, and higher blood iCa concentration cannot be simply equated with a superior control strategy. The core mechanism by which clinoptilolite prevents postpartum hypocalcemia is to moderately reduce maternal calcium and phosphorus absorption prepartum, allowing cows to adapt in advance to the sharp increase in calcium demand at the onset of lactation. Accordingly, the lower blood iCa concentration in the CLN group during the prepartum period was consistent with its regulatory mechanism.

At calving, we observed that although the mean blood iCa concentration was lower in the CLN group, the dispersion of blood iCa concentrations was smaller. No severe hypocalcemia (iCa ≤ 0.85 mmol/L) was found in the CLN group, whereas such cases occurred in the DCAD group, which had a higher mean blood iCa concentration prepartum. The wider dispersion of blood calcium at calving in the DCAD group may be related to individual differences in feed intake caused by the poor palatability of anionic salts. Inconsistent feed intake not only affects calcium intake but also interferes with postpartum care efficacy: cows with insufficient feed intake develop hypocalcemia due to low calcium supply, while cows with normal feed intake and high baseline blood calcium may activate calcitonin negative feedback regulation after postpartum calcium supplementation.

Following oral calcium administration, the number of cows with subclinical hypocalcemia at 1 h postpartum decreased from 6 to 4 in the DCAD group and from 8 to 1 in the CLN group. The rapid recovery of blood calcium in the CLN group may be attributed to reduced blood phosphorus, which synergistically promotes bone resorption. As reported by Wächter et al. [23], cows fed a low-phosphorus diet prepartum exhibited higher bone resorption marker levels and faster blood calcium recovery postpartum. Despite lower blood iCa at calving, the CLN group achieved quicker blood calcium recovery through the combined effects of low calcium and low phosphorus.

At 24 h postpartum, blood calcium concentrations returned to normal in all 10 cows in the DCAD group. In the CLN group, 2 cows remained hypocalcemic: 1 with persistent hypocalcemia and 1 with mild subnormal calcium (1.03 mmol/L). Neither of these two cows developed parturient paresis.

### 4.2. Changes in PTH

According to the classic theory of calcium homeostasis regulation, blood calcium in pregnant and lactating dairy cows is primarily maintained by PTH. Findings from the present study suggest that both prepartum dietary strategies can increase postpartum blood calcium concentrations in dairy cows, and their regulatory patterns may not fully align with the classic theory.

During the final trimester of gestation, approximately 85% of total calf body calcium is deposited in the fetal skeleton, with calcium demand rising sharply from around 2.3 g/d at d 190 of gestation to approximately 10.3 g/d by d 280 [24]. Meanwhile, the mammary gland begins secreting colostrum approximately 5 weeks prepartum [25], which contains 1.7–2.3 g/L of calcium, further imposing a substantial calcium requirement.

Under the classic model, cows would be expected to secrete higher levels of PTH at this stage to enhance calcium mobilization from bone and absorption from the gastrointestinal tract, thereby meeting the demands of fetal mineralization and colostrogenesis. Contrary to this expectation, we observed a declining trend in serum PTH concentrations in both treatment groups during the prepartum period, reaching the lowest level at parturition (0 h). Similarly, Seely et al. demonstrated in a previous study that serum PTH concentrations consistently decreased from 5 d prepartum to 1 d prepartum in all cows, regardless of whether they developed transient, persistent, or delayed subclinical hypocalcemia [26].

This pattern of prepartum PTH changes in dairy cows shows similarities to that in humans. In pregnant women, serum PTH concentration gradually declines from early gestation and becomes nearly undetectable at term, whereas parathyroid hormone-related protein (PTHrP) rises progressively and peaks postpartum [27]. One possible explanation is that PTH plays only a secondary regulatory role during pregnancy and lactation in cows.

In some women from Asia and Africa with insufficient calcium or vitamin D intake, or with restricted calcium absorption due to high phytate consumption, serum PTH increases compensatorily and remains normal or elevated [28,29,30]. In contrast, dairy cows have much higher calcium requirements than humans and most other mammals: not only is milk calcium concentration higher, but milk yield far exceeds the needs of fetal growth [9]. During the final week prepartum, cows are expected to be in a relative calcium-deficient state, and PTH secretion should be stimulated to increase. As some researchers have proposed, PTHrP plays a non-negligible role in calcium homeostasis during lactation in dairy cows [31,32].

In the present study, the consistent decline in serum PTH during the prepartum period in both groups may reflect that mammary-derived PTHrP partially replaces the calcium-regulatory function of PTH. A recent study by Frizzarini [18] supports this conclusion: although both zeolite A and anionic salt treatments increased postpartum blood calcium, neither treatment altered PTH concentrations statistically compared with controls, either pre- or postpartum. However, both groups exhibited significantly higher serum serotonin concentrations than controls during the 3 days prepartum. Previous research has confirmed that serotonin can elevate blood calcium via the serotonin–PTHrP–calcium axis [33].

Secondly, at −14 d prepartum and at 0 h postpartum, cows in the DCAD group presented significantly higher concentrations of serum PTH than those in the CLN group, despite having higher blood calcium concentrations (*n* = 50). This difference may be related to blood acid–base status and serum phosphorus levels. Extracellular pH regulates PTH secretion by modulating the calcium-sensing receptor (CaSR) on parathyroid chief cells [34]. Acidosis (pH = 7.2) blunts the sensitivity of the CaSR, which promotes PTH release, whereas alkalosis (pH = 7.6) enhances CaSR activity and suppresses PTH. Serum phosphorus levels also modulate this pathway; concentrations above 2.0 mM inhibit CaSR via non-competitive antagonism, further stimulating PTH secretion [35]. Since clinoptilolite significantly lowers serum and salivary phosphorus [18], it likely relieves this inhibition, restoring CaSR sensitivity and keeping PTH levels lower than those seen in the DCAD group. This aligns with findings in low-phosphorus models where bone resorption increases without upregulating PTH gene expression, likely due to the suppression of bone-derived FGF23 [36,37].

During the postpartum period, blood calcium and PTH concentrations were generally similar between groups, except at 24 h when DCAD cows had higher iCa. Notably, we observed a partial “decoupling” of the classical feedback loop, as DCAD cows exhibited both elevated iCa and elevated PTH at this time point. This mirrors Frost et al. (2024) [38], who found that PTH remained high in fresh cows regardless of their calcium status, suggesting the standard inverted “S-shaped” regulation shifts during lactation. The physiological set-point for PTH likely moves upward to support the intense calcium demand of milk synthesis. Similar adaptations occur in lactating rats, where parathyroid cells become less sensitive to inhibitory signals and continue secreting high PTH even when blood calcium is elevated ([39,40]). Therefore, the deviation we observed likely stems from a lactation-specific adaptive modification of the parathyroid chief cells.

### 4.3. Periparturient Metabolic Changes

The transition period forces dairy cows into a state of metabolic reprogramming to handle lactation. Our data show that the two prepartum strategies shape this network differently, primarily by altering systemic acid–base balance.

The DCAD diet worked by inducing metabolic acidosis. At calving (0 h), 35.0% of DCAD cows were acidotic, compared to just one cow (1.7%) in the CLN group. This acidosis has pros and cons. It can enhance the sensitivity of PTH receptors in organs such as bone, intestine, and kidney, and avoids the impacts of metabolic alkalosis on the conformation of PTH receptors and downstream calcium regulatory pathways. Postpartum cessation of the anionic diet resulted in a rapid, V-shaped recovery of blood pH, replicating patterns described by Zhang et al. [41]. Conversely, the CLN group maintained a stable, marginally elevated blood pH. This physiological pattern is likely attributable to the natural prepartum depression in dry matter intake and was rapidly corrected following the resumption of feed consumption postpartum.

Acid–base shifts are closely linked to electrolyte balance. Even though the CLN group consumed more dietary potassium (1.78% vs. 1.49%), their blood potassium levels remained lower than the DCAD group (though still within normal range). This aligns with [18], who used synthetic zeolite A. Similarly, zeolite-based hemostatic agents have been shown to lower blood Na^+^ and K^+^ in humans [42]. We suspect that clinoptilolite acts as a cation exchanger in the rumen [43], binding dietary potassium before it can be absorbed.

Conversely, the higher blood potassium in the DCAD group reflects a conflict between two forces: the dietary restriction of potassium versus the acidosis-induced shift of intracellular potassium into the blood. This complicates management. While producers feed low-potassium forages (like oat hay) to control intake, excessive acidification can drive blood potassium up anyway. High blood potassium is strongly alkalinizing and can cause PTH resistance, potentially canceling out the benefits of the anionic diet.

Energy metabolism is another piece of the puzzle. While some studies suggest acidosis impairs insulin sensitivity (raising glucose) [44] or lowers glucose by suppressing feed intake [45], we found no difference in prepartum glucose levels. This is likely because our moderate DCAD formulation did not severely impact dry matter intake. The stable glucose dynamics in the CLN group may also stem from clinoptilolite’s ability to stabilize the rumen environment and optimize VFA production [21].

These metabolic differences explain the differences in the status of calcium at calving. The mild acidosis in the cows in the DCAD group primes their bone and kidney tissues to respond to PTH [46], allowing them to maintain higher iCa and PTH levels during the calcium drain of parturition. As a result, all cows in the DCAD group recovered normal calcium levels within 24 h. The CLN group, operating without acidosis, probably relied on indirect pathways, such as intestinal phosphate binding and the phosphate-FGF23/serotonin-PTHrP axes [33]. The activation of this mechanism was slower at the critical moment of calving, which caused a few cows to develop subclinical hypocalcemia at 24 h. These cows had normal pH and potassium levels, which ruled out electrolyte imbalances as the cause.

## 5. Conclusions

Prepartum phase anionic, salt diets significantly elevated blood iCa and PTH levels in periparturient dairy cows by inducing metabolic acidosis, yet inevitably caused drastic fluctuations in blood pH and serum K^+^ concentrations. In contrast, cows in the CLN group maintained stable blood pH and electrolyte balance throughout the prepartum phase, confirming that clinoptilolite—as a non-acidifying regulatory agent—avoids the metabolic stress associated with anionic salts and provides a milder, safer transition for dairy cows. Although blood calcium concentrations were lower in the CLN group at calving, they returned to normal levels more rapidly when combined with postpartum calcium supplementation.

Under both dietary regulation strategies, blood PTH levels in prepartum cows showed a decreasing trend, indicating that the classic PTH-mediated calcium homeostasis regulation theory is not fully applicable during the periparturient period, and other hormones such as PTHrP may be involved. Postpartum cows presented partial “uncoupling” between serum calcium and PTH concentrations: PTH levels remained high even when serum calcium concentrations increased. This finding suggests that the calcium-sensing threshold of the parathyroid gland in lactating cows may be upregulated, and the adaptive changes in calcium homeostasis regulation require further investigation.

## Figures and Tables

**Table 1:** Ingredient and nutrient compositions of the experimental diets.

Item	CLN-Pre	DCAD-Pre	CLN-Post	DCAD-Post
Ingredient Composition, % of DM ^1^				
Oat hay	5.50	4.80	1.10	0.50
Alfalfa hay	—	—	2.30	—
Alfalfa silage	—	—	1.00	6.00
Corn, ground	0.89	—	3.98	—
Soybean meal	0.33	—	3.24	—
Concentrate supplement	1.14	3.00	2.23	7.12
Other ingredients ^2^	27.14	22.50	19.44	18.49
Nutrient Composition, % of DM				
Dry matter (%)	49.00	47.30	50.50	50.89
Crude protein	15.00	15.80	17.50	17.48
Acid detergent fiber	25.80	25.43	17.00	16.58
Neutral detergent fiber	45.90	40.1	27.50	25.20
Lignin	5.40	9.9	3.60	3.30
Ether extract	2.76	2.70	5.43	4.30
Starch	16.50	19.21	26.00	26.90
Non-fiber carbohydrates	26.70	34.30	40.65	44.70
Ash	7.65	8.18	9.10	11.69
Calcium	0.49	0.70	1.10	1.20
Phosphorus	0.43	0.37	0.45	0.40
Potassium	1.78	1.49	1.62	1.63
Magnesium	0.38	0.43	0.40	0.45
Sodium	0.14	0.15	0.63	0.61

^1^ Values are expressed as a percentage of dry matter (DM) unless otherwise noted. CLN = diet supplemented with clinoptilolite; -DCAD = negative DCAD diet. Pre = diet from 21 days before calving to parturition; Post = diet after parturition. The DCAD of the -DCAD-Pre diet was −10.7 mEq/100 g DM. ^2^ Other ingredients include water, wheat straw, wheat bran, rapeseed meals, cottonseed products, beet pulp, molasses, extruded soybean, and steam-flaked corn, not listed separately; “—” indicates that the ingredient was not included in the diet or that the data were not available.

**Table 2:** Prepartum distribution of blood iCa in the DCAD and CLN groups.

Group\iCa Concentration (mmol/L)		−21 d	−14 d	−7 d	0 h
DCAD (*n* = 50)	≤0.84	0	0	0	0
	0.85–1.05	0	0	0	13
	≥1.06	50	50	50	37
CLN (*n* = 50)	≤0.84	0	0	0	0
	0.85–1.05	0	0	0	31
	≥1.06	50	50	50	19

**Table 3:** Distribution of blood iCa concentrations while calving in cows from the DCAD and CLN groups.

Group\iCa Concentration (mmol/L)		0 h	1 h	6 h	12 h	13 h	24 h
DCAD (*n* = 10)	≤0.84	1	0	2	0	0	0
	0.85–1.05	5	4	2	3	2	0
	≥1.06	4	6	6	7	8	10
CLN (*n* = 10)	≤0.84	0	0	0	0	0	0
	0.85–1.05	8	1	2	1	1	2
	≥1.06	2	9	8	9	9	8

## Data Availability

The original contributions presented in this study are included in the article Further inquiries can be directed to the corresponding authors.

## Abbreviations

The following abbreviations are used in this manuscript:

ANCOVA	Analysis of covariance
BCS	Body condition score
CaSR	Calcium-sensing receptor
CLN	Clinoptilolite
DCAD	Dietary cation-anion difference
DM	Dry matter
FGF23	Fibroblast growth factor 23
Glu	Glucose
IACUC	Institutional Animal Care and Use Committee
iCa	Ionized calcium
NEB	Negative energy balance
NEFA	Non-esterified fatty acid
PTH	Parathyroid hormone
PTHrP	Parathyroid hormone-related protein
SCH	Subclinical hypocalcemia
SOP	Standard operating procedure
TMR	Total mixed ration
